# An Update on Protein, Leucine, Omega-3 Fatty Acids, and Vitamin D in the Prevention and Treatment of Sarcopenia and Functional Decline

**DOI:** 10.3390/nu10081099

**Published:** 2018-08-16

**Authors:** Anne-Julie Tessier, Stéphanie Chevalier

**Affiliations:** 1 School of Human Nutrition, McGill University, 21111 Lakeshore Rd, Ste-Anne-de-Bellevue, QC H9X 3V9, Canada; anne-julie.tessier@mail.mcgill.ca; 2 Research Institute of the McGill University Health Centre, 1001 Décarie Blvd, Montreal, QC H4A 3J1, Canada; 3 Department of Medicine, McGill University, 845 Sherbrooke St. W, Montreal, QC H3A 0G4, Canada

**Keywords:** protein, leucine, vitamin D, omega-3 fatty acids, sarcopenia, muscle strength, physical performance, older adults, frailty

## Abstract

Aging is associated with sarcopenia and functional decline, leading to frailty and disability. As a modifiable risk factor, nutrition may represent a target for preventing or postponing the onset of these geriatric conditions. Among nutrients, high-quality protein, leucine, vitamin D, and omega-3 polyunsaturated fatty acids (*n*-3 PUFA) are of particular interest for their demonstrated effects on skeletal muscle health. This narrative review aims to examine the recent observational and interventional evidence on the associations and the role of these nutrients in the muscle mass, strength, mobility, and physical function of free-living older adults, who are either healthy or at risk of frailty. Recent evidence supports a higher protein intake recommendation of 1.0–1.2 g/kg/day in healthy older adults; an evenly distributed mealtime protein intake or minimal protein per meal may be beneficial. In addition, vitamin D supplementation of 800–1000 IU, particularly when vitamin D status is low, and doses of ~3 g/day of *n*-3 PUFA may be favorable for physical function, muscle mass, and strength. Reviewed studies are highly heterogenous, yet the quantity, quality, and timing of intakes should be considered when designing intervention studies. Combined protein, leucine, vitamin D, and *n*-3 PUFA supplements may convey added benefits and may represent an intervention strategy in the prevention of sarcopenia and functional decline.

## 1. Introduction

Sarcopenia is defined as the generalized and progressive loss of muscle mass and strength [1,2], leading to declines in physical function and mobility. These are integral components of frailty defined by a decrease in the function of several physiological and psychological systems, which increases vulnerability to stressors [2]. In addition to reducing the quality of life [3], these age-related conditions increase risks of morbidity [4,5], hospitalization [6] and its associated costs [7], and mortality [8]. Sarcopenia has a multifactorial etiology, namely neuromotor dysfunction, chronic low-grade inflammation, physical inactivity, decreased endocrine function, and poor nutritional status [1]. The latter, resulting from a reduction in dietary intake among other causes, was previously associated with lower physical function and frailty among older adults from various settings (hospitals, community, rehabilitation) [9,10,11]. Approximately two-third of older adults are estimated as malnourished or at risk of becoming malnourished [12]. Adding to a poor nutritional status in a context of advanced age is the presence of anabolic resistance, which is characterized by a reduced response to anabolic stimuli including dietary protein, leading to higher protein needs compared to those of younger adults [13].

**Figure 1:**
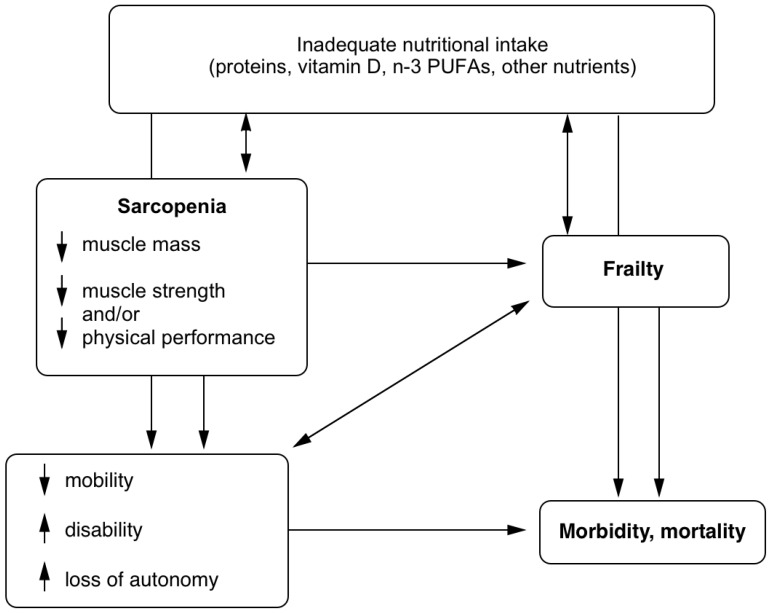
Potential role of nutrition on the physical health of older adults. Short arrows within boxes: increase or decrease. Long arrows between boxes: may lead to. Double-sided arrows: the relationship may be bidirectional. Arrows passing through boxes: factors in box could be mediators.

As a modifiable risk factor, nutrition is a potential target to improve or prevent the loss of physical function in older adults (Figure 1). Specific nutrients are of particular interest for their demonstrated role on the muscular system, and have been the object of earlier and more recent studies, either as single supplements or in combination with other supplements. These include proteins, especially those rich in leucine, which is the most potent branched-chain amino acid at stimulating muscle protein synthesis (MPS) [14], vitamin D, and *n*-3 polyunsaturated fatty acids (*n*-3 PUFAs). This narrative review aims to examine the latest observational and interventional (supplementation alone, without physical exercise) evidence on the associations and the role of these nutrients in the muscle mass, strength, mobility, and physical function of free-living older adults, who are either healthy, frail, or at risk of functional decline.

## 2. Proteins and Amino Acids

### 2.1. Total Dietary Protein Intake

Inadequate dietary protein intake is generally recognized as an etiologic factor contributing to sarcopenia [1]. In an effort to prevent sarcopenia and maintain physical function and long-term optimal health, in 2013 and 2014, large international groups of experts issued a consensus to increase protein recommendations to 1.0–1.2 g/kg/day for healthy individuals, 1.2 g/kg/day for active individuals, and 1.2–1.5 g/kg/day for those with chronic or acute diseases (except renal) [15,16] from the current recommended daily allowance (RDA) of 0.83 g/kg/day [17]. This consensus was based on substantial evidence emanating from metabolic, interventional, and observational studies that were then available, and continues to be supported by more recent evidence, comprehensively reviewed elsewhere [18]. In brief, metabolic studies concur to show greater requirements than previously estimated by nitrogen balance, and the majority of observation studies concur to show higher lean or muscle mass and strength and lesser risk of losses and functional decline in subgroups of the older population consuming more dietary protein.

*Interventional studies.* On the other hand, randomized clinical trials of protein or amino acid supplements have reported opposing results on muscle mass or strength. From nine trials included in a recent meta-analysis, no significant effect of supplements was found on lean body mass, leg strength, or handgrip strength [19]. Heterogeneity was evident for trials that differed in the studied population (healthy, frail, diabetic, or sarcopenic individuals), duration, and supplement forms and doses. Importantly, usual dietary protein intake was not measured in all of the studies, therefore limiting data interpretation as to additional protein effect. The issue of compliance, which is difficult to measure with precision, is also to be considered, since these supplements are not always palatable and become monotonous over long periods of time. In that respect, Mitchell et al. (2017) conducted a well-controlled feeding study of 35 healthy older men (>70 years old), providing all prepared foods to test a diet at the current protein RDA against twice the RDA, i.e., 0.8 g/kg/day or 1.6 g/kg/day, for 10 weeks [20]. While the appendicular lean mass (ALM) remained unchanged in individuals following the 1.6 g/kg/day diet, it decreased in the RDA group. This strengthens the higher protein needs for the maintenance of muscle mass with aging. With regards to functional outcomes, benefits in favor of supplements versus placebo were reported as increased grip strength [21], improvement in functional limitations [22], the absence of deterioration [23], or an improvement [24] in physical performance (Short Physical Performance Battery score, SPPB) whereas no improvement was observed in others [20,25].

Again, this general lack of effect of protein supplements as opposed to positive associations between protein intake and muscle mass and function observed in cohort studies could be due to the type, dose, and timing of supplements, as well as compliance, and the population studied. Indeed, from the above studies, beneficial effects of protein supplements on physical function appear to be seen especially in frail, malnourished individuals or those at risk of malnutrition.

### 2.2. Meal Distribution of Dietary Protein

Beyond total daily protein intake, the effect of its mealtime distribution is a topic of emerging interest in the field of sarcopenia research. To equally distribute protein intake across the three daily meals is based on the concept of reaching a per-meal anabolic threshold [26,27]. A combination of factors such as insulin resistance [28,29,30], sedentary lifestyle or short-term immobilization [31,32], inflammation, lipotoxicity, and oxidative stress [33] are thought to cause anabolic resistance [27,33]. An elevated anabolic threshold translates into greater needs to achieve maximal stimulation of MPS in older compared to younger individuals [34,35,36]. Earlier studies agreed upon a desirable dose of 25–30 g of high-quality protein per meal, providing ≥15 g of essential amino acids, which are recognized as the main amino acids responsible for stimulating MPS [37].

*Interventional studies.* The even distribution hypothesis was first confirmed in a crossover study in young adults (*n* = 8) comparing a seven-day diet of evenly (30/30/30 g) versus unevenly (11/16/63 g) distributed protein intake. MPS measured by incorporation of ^13^C_6_-phenylalanine over 24 h was significantly higher following the diet with an even distribution [38]. In contrast, a randomized trial testing the distribution at two levels of intake (0.8 g/kg/day and 1.5 g/kg/day) over four days in older adults (52–75 years old) concluded that quantity of dietary protein intake, but not distribution, resulted in an increased MPS and net protein balance [39]. These studies were of too short duration to demonstrate effects on muscle mass or strength. To date, only the longer-term intervention study (42 days) testing distribution has been conducted in malnourished hospitalized elderly patients. They consumed on average 1.3 g/kg/day from a diet that provided either >70% of proteins at lunch (bolus) or an amount that was divided into four meals (spread; *n* = 30–36/group). Lean body mass significantly increased in the group that was fed the protein bolus diet. No change in grip strength was reported in neither of the groups [40]. It is possible that none of the four meals in the spread diet provided enough protein to reach the anabolic threshold, which would theoretically be higher in these malnourished and inactive patients. Clearly, the response to protein intake may differ according to health status, which justifies further research in vulnerable persons at risk of malnutrition.

*Observational studies.* We examined the loss of lean mass related to protein intake distribution using data from 351 and 361 free-living men and women aged 67–84 years of the Quebec Longitudinal Study on Nutrition and Successful Aging (NuAge) [41]. We found that a more evenly distributed protein intake, regardless of quantity, was associated with higher lean mass at baseline and two-year follow-up after adjustment for relevant covariates [42]. However, neither total intake nor protein distribution were related to the rate of loss over the two-year follow-up, which is a period that was perhaps not long enough to detect association with a marginal decline (2% across two years). Muscle strength and mobility were also studied in this cohort (*n* = 1741; three-year follow-up). A composite score was created for each of these parameters, embedding handgrip, arm strength, and leg strength, and timed-up-and-go (TUG), chair-stand, and walking speed, respectively. In both sexes, a more evenly distributed protein intake was positively associated with muscle strength throughout the study, but not with mobility, and did not predict strength or mobility rate of decline [43]. A recent and smaller cross-sectional study (*n* = 140) reported a positive association between a more even protein distribution and gait speed, but not with total SPPB and its other single components. Muscle mass and strength were not measured [44]. Finally, no such associations were found in a small cross-sectional study (*n* = 99) of successful agers. However, these results did not represent a broad range of functional capacity, thus pointing to dietary protein intake and distribution perhaps having more impact on those with impaired muscle health [45]. Since both total and mealtime distribution of protein may influence muscle mass and function, a per-meal minimal intake has been advocated [46]. Testing this rationale in 4123 adults >50 years old in the 2011–2014 National Health and Nutrition Examination Survey (NHANES), grip strength was positively associated with consumption of ≥25 g protein/meal on two or more eating occasions compared to only one eating occasion of the same amount. This relationship disappeared when adjusted for multiple covariates, including total daily protein intake, which was related with grip strength [47]. In contrast, in 1081 older adults (50–85 years) of the 1999–2002 NHANES, Loenneke et al. (2016) found that participants consuming at least two meals containing 30–45 g protein/meal had the greatest leg lean mass and strength, and those consuming at least one meal/day of ≥30 g protein/meal had greater responses than those with no meal reaching the 30 g threshold [48]. Adjusted models included age, sex, ethnicity, smoking, physical activity, total carbohydrate intake, total fat intake, and relative protein intake (g/kg). It appears that a threshold of 30 g versus 25 g may explain discrepancies between studies, but it was more likely that lower limb muscles respond more to protein intake due to solicitation for mobility than handgrip strength.

In summary, in healthy older adults, mealtime protein distribution may have more of an impact when other anabolic stimuli are minimal, i.e., at low total protein intakes and physical activity levels, which remains to be investigated in longer-term intervention studies. To date, per-meal protein doses sufficient to generate anabolism in persons at risk of malnutrition and sarcopenia are still unknown.

## 3. Leucine

Leucine is the most potent amino acid to stimulate MPS from activation of the nutrient and growth factor-sensing mammalian target of rapamycin complex 1 (mTORC1) and in turn, its downstream targets ribosomal protein S6 kinase 1, translation initiation factor 4EBP-1, and elongation factor 2 [49,50]. Though not fully elucidated, mTORC1 activation by leucine is thought to occur at the lysosome through a cascade involving Ragulator Rag GTPases and vacuolar H+-ATPase [51,52]. Three to fourfold increments in plasma leucine appear to be required to elevate intracellular concentrations and augment MPS [53] provided that other essential amino acids are also available to sustain greater protein synthesis [54].

*Interventional studies*. Very few cohort studies have associated dietary leucine intake to muscle mass. Since leucine is ubiquitously found in all proteins, though in animal more than plant proteins, its intake is practically impossible to dissociate from total protein intake from foods. Thus, most of the evidence on leucine’s effect has been accrued from supplement studies, most of which were of short-term duration. Two meta-analyses published in 2015 concluded differently [55,56]. The one from Xu et al. (2015) included nine randomized controlled trials (RCTs), four testing acute post-challenge responses (three in healthy, one in cancer participants), and five longer-term, ranging from 10 days to six months (in participants who were either healthy, had type 2 diabetes, or had polymyalgia, and were on bed rest, or exercising) [56]. A pooled effect of 1.08 (95% CI: 0.50–1.67) was found on acutely increasing MPS, but no effect was observed on lean body mass or leg lean mass from longer-term interventions. The meta-analysis by Komar et al. (2015) included 16 studies testing leucine-rich supplements in a wider variety of participants, who were also frail, sarcopenic, and institutionalized [55]. Subgroup analysis revealed a mean effect of 1.14 kg (95% CI: 0.55–1.74) increase in lean body mass in favor of leucine-rich supplements in sarcopenic, but not in healthy participants. In both meta-analyses, leucine was either given as pure crystalline powder, or as part of essential amino acid drinks, complete medical formula, or whey protein, at doses ranging from 2 g/day to 17.6 g/day. This considerable heterogeneity in population, study design, and the type of supplements studied precludes firm conclusions, but points to plausible effects in persons having or at risk of sarcopenia. The question as to why the acute stimulation of MPS by leucine supplements does not seem to translate into measurable changes in lean body mass in healthy older adults remains open. Insufficient usual dietary protein intake, poor long-term compliance, and perhaps habituation to a sustained stimulus may explain negative findings.

More recent studies from Phillips et al. have revived a promising anabolic role for leucine [57,58,59]. Using an integrative measure of myofibrillar protein synthesis (MyoPS) over three days in well-controlled crossover feeding studies, providing all foods to older men, 5 g of leucine that was added to each of the three daily meals resulted in augmented MyoPS in both the rested leg and the one submitted to unilateral resistance exercise [59]. Interestingly, this effect was seen at both low (0.8 g/kg/day) and higher (1.2 g/kg/day) daily protein intakes. In healthy older women, 10 g of whey protein added with 3 g of leucine were compared to 25 g of whey protein intrinsically containing 3 g of leucine, taken twice daily for six days. Results showed that the lower protein, leucine-matched supplement was as effective as the 25-g protein dose at increasing acute and integrated MyoPS, which could represent a practical alternative for older women with typical low appetite [57]. Lastly, Devries et al. (2018) tested the twice daily consumption of 15 g of a milk protein drink containing 4.2 g of leucine against 15 g of mixed protein drink containing 1.3 g of leucine, as part of a diet providing 1 g protein/kg/day, under the same protocol in older women [58]. Greater acute postprandial and integrated MyoPS responses over six days were found with the higher leucine-containing drink. Altogether, these positive results obtained in rigorous conditions are promising and warrant corroboration in longer-term interventions to demonstrate potential benefits of leucine for preserving muscle mass and function.

## 4. Vitamin D

### 4.1. Vitamin D, Physical Function, and Muscle Mass and Strength

Vitamin D is a key nutrient in musculoskeletal health. In adults, vitamin D deficiency is associated with bone diseases including osteomalacia, osteopenia, and osteoporosis, and increases the risk of fractures [60]. However, bone health is not the only physiological dimension to be impacted by vitamin D, since its involvement has been evidenced in cardiovascular disease, autoimmune diseases, and cancer prevention [61], among others. There has been growing interest in the implications of vitamin D status in the physical function of older adults given the high prevalence of vitamin D deficiency in this population [62]. The ubiquity of vitamin D receptors (VDR) in various tissues, including muscles, is well recognized [63]. From its binding to VDRs, vitamin D mediates genomic and non-genomic effects in muscle cells; it namely promotes muscle contractility through calcium influx, myoblast differentiation, and the insulin sensitivity of muscles [64]. The current RDA for vitamin D intake is 600 IU/day for persons aged 1–70 years, and 800 IU/day for older adults (≥71 years), which translates into serum 25-hydroxyvitamin D (25(OH)D) level ≥50 nmol/L for skeletal health [65].

*Observational studies*. While large cross-sectional studies corroborate a relationship between insufficient level of serum 25(OH)D (<50 nmol/L) and low physical performance [66,67,68,69,70,71], mobility [66,68,69,70], muscle strength [66,67,69,70,72,73], and greater disability [66,73] in free-living older adults, the association with muscle strength was not found in a cohort of older women (>90% with vitamin D insufficiency) [74]. In 2017, a meta-analysis of 17 cross-sectional and five longitudinal studies (*n* = 54–4100) provided fair evidence that seniors with low vitamin D status, regardless of the cut-point used for its definition, had a slower usual gait speed compared to those with normal status (−0.18 m/s in vitamin D deficient; ≤25 nmol/L) [75]. Physical performance (by TUG) was also associated with vitamin D deficiency. More recently, Vaes et al. (2018) confirmed cross-sectional associations between vitamin D insufficiency, gait speed (*n* = 745), and TUG (*n* = 488) in older men and women aged ≥65 years; interestingly, frail individuals were at higher risk of being vitamin D insufficient compared to non-frail individuals [76], which is an association that has also been ascertained in a meta-analysis [77]. No link with muscle strength was found in this cohort.

Vitamin D insufficiency has also been longitudinally associated with greater risks of disability [69,78,79], decline in physical performance [71,80], and handgrip strength [81] in healthy older adults. However, few groups did not find such associations (*n* = 988–2099; 2.5 to six years follow-up) [69,82]. Since then, Granic et al. (2017) studied very old adults (*n* = 845, age ≥85 years old) and found a greater handgrip strength decline in men of the lowest 25(OH)D season-specific quartile over five years, but this was not seen in women [83]. Although causal effect cannot be concluded from these observational studies, altogether the evidence suggests that interventions should aim at targeting at risk populations, namely individuals with vitamin D insufficiency or deficiency, and frail seniors to favor better mobility or delay the onset of disabilities.

*Interventional studies*. Systematic reviews and meta-analyses examined the benefits of vitamin D supplementation on physical performance, muscle mass, and strength in community and/or institution-dwelling seniors [84,85,86,87]. The importance of considering baseline serum 25(OH)D concentrations has been emphasized, since individuals with vitamin D deficiency appear to be more responsive to supplementation [84]. Modest mean differences in TUG (3 studies; *n* = 551) and postural sway (three studies; *n* = 413) independent of doses were reported following vitamin D supplementation in a first meta-analysis; yet authors observed that all of the studies providing high daily vitamin D doses (i.e., 800 IU to 1000 IU) supported beneficial effects on balance and lower extremity muscle strength [85]. A second meta-analysis’s age subgroup analysis showed a favorable effect of vitamin D supplementation, with or without calcium, on muscle strength in older adults ≥65 years old and especially, greater improvement in institutionalized compared to free-living individuals [87]. In line with these previous studies, the most recent meta-analysis of RCTs (2017) conducted in community-dwelling older adults confirmed a slight improvement of −0.3 s (95% CI: −0.1 to −0.5; 5 studies; *n* = 1260) in the TUG test following supplementation, but no overall increase in handgrip strength was detected (seven studies; *n* = 1452) [86]. The included RCTs assessing TUG performance provided doses ranging from 800 IU to 2000 IU/day between 10 weeks and 20 months, and one study provided 150,000 IU every three months for a nine-month period. The latter revealed an effect in favor of the placebo [88], and no effect was found from 2000 IU/day for 10 weeks [89]. Further, one additional study by Bischoff-Ferrari et al. (2016) that was not included in the abovementioned meta-analysis established a null effect of monthly doses of 24,000 IU + calcifediol and 60,000 IU on lower extremity function (by SPPB) after one year, and led to higher fall incidence compared to 24,000 IU/month [90]. This finding strengthens that benefits are observed with vitamin D doses within the range of 800–1000 IU/day, but not necessarily at higher doses.

Heterogeneity between RCTs (doses and type of supplement, duration of the intervention, participants’ baseline vitamin D status) and few discrepancies among meta-analyses with regard to selection criteria makes comparison between studies difficult. Nonetheless, in light of the pooled evidence, vitamin D supplementation should be considered to improve physical performance in older adults and perhaps for muscle strength in most likely frail seniors.

### 4.2. Vitamin D and Fall Prevention

Unintentional falls are the leading cause of injury death in seniors aged ≥65 years in the United States (US) [91]. Beyond musculoskeletal-related functions, risk factors for falls belong to multiple intrinsic (biological) and extrinsic (socio-economic, environmental, and behavioral) dimensions; these include balance, gait abnormalities, chronic conditions such as neurological disorders, cognitive impairment, vision, fear of falling, use of medications, and environmental hazards, all of which contribute to the occurrence of falls [92].

*Interventional studies*. Vitamin D and falls is an area that has been extensively studied; trials have tested the impact of vitamin D supplementation on the prevention of falls and numerous meta-analyses have examined the overall reported effects. However, due to substantial differences in selection criteria between meta-analyses [93], conflicting results remain. In their high-quality meta-analysis of 14 trials and 27,522 participants, Bolland et al. reported a non-meaningful effect of vitamin D, with or without calcium, on the relative risk (0.96; 95% CI: 0.91–1.01), and highlighted that it would unlikely change with additional future studies [94,95].

In 2018, an updated evidence report and systematic review of fall prevention interventions in community-dwelling older adults was published for the US Preventive Service Task Force. From the seven studies of vitamin D supplementation included in the review, the authors found inconsistent results, which was possibly due to high heterogeneity [96]. Also, one trial in older women showed the deleterious effects of an annual 500,000 IU vitamin D3 dose on falls and fractures [97]. Following this report, the new recommendation advises against vitamin D supplementation for fall prevention in community-dwelling non-osteoporotic and non-vitamin D deficient older adults ≥65 years old [98].

## 5. N-3 Polyunsaturated Fatty Acids

N-3 PUFAs are consumed as eicosapentaenoic acid (EPA; 20:5 *n*-3) and docosahexaenoic acid (DHA; 22:6 *n*-3) or as alpha-linolenic acid (ALA; 18:3 *n*-3), of which a very limited fraction is converted to EPA (8 and 21% conversion rates) and DHA (~0 and 9%) in men and women, respectively [99,100]. N-3 PUFAs are well-known for their anti-inflammatory properties and their role in the development and maintenance of neurocerebral functions [101,102,103]. In a large prospective cohort study of older adults, plasma *n*-3 PUFA levels were associated with a 27% reduction in total mortality risk across quintiles, increasing the life expectancy of individuals in the highest quintile by ~2 years [104]. This association with mortality was essentially ascribed to docosapentaenoic acid (DPA; 22:5 *n*-3), DHA, and EPA status, and was more pronounced for cardiovascular disease mortality, including coronary heart disease and ischemic stroke. No RDA recommendations exist for *n*-3 PUFA, only adequate intake (AI) was established for ALA (1.6 g/day for men and 1.1 g/day for women, aged ≥14 years) [17]; however, the majority of expert groups endorse intakes of 250–500 mg/day EPA and DHA for cardiovascular health, which translates into ~2 servings (140 g, 5 oz) of fatty fish per week [105]. Although older adults tend to consume more fish that are high in n-3 PUFA compared to younger adults, their intake remains suboptimal, with a mean of 0.19 ± 0.02 oz/day [106]. Although still elusive, the anabolic role of n-3 PUFAs on skeletal muscle is thought to be owed to a reduction in pro-inflammatory cytokines, myosteatosis, an improvement of insulin sensitivity [107], the stimulation of muscle protein synthesis via the mTOR-p70S6k signaling pathway [108], and a diminution of mitochondrial reactive oxygen species emission [109].

*Observational studies.* Inconsistent associations were found between *n*-3 PUFA dietary intakes and muscle function from scarce cross-sectional evidence [110,111], and to our knowledge, no study has examined this relationship since then. Previous evidence showed higher plasma *n*-3 PUFA levels, which is an objective biomarker of dietary intake [112], to be associated with better physical performance and gait speed in healthy older adults at baseline and with a lower risk of poor physical performance (odds ratio, OR = 0.21, 95% CI: 0.08–0.53; *n* = 884) over three years (relationship owing to EPA and DHA) [113]. This cross-sectional association with gait speed was also confirmed recently [114]; however, one study did not find red blood cell membrane EPA and DHA levels to be associated with physical performance in participants at risk of cognitive decline after controlling for covariates [115]. As for muscle strength, the relationship is unclear, as evidenced by one study reporting no association with plasma *n*-3 PUFA once adjusted for covariates [116].

Longitudinal analyses with follow-up periods ranging from three to five years were performed on the cohorts cited above. One study found baseline total plasma *n*-3 PUFAs and DHA to be associated with incidences of self-reported mobility disability after five years in healthy older women only (OR = 0.48, 95% CI: 0.25–0.93), suggesting that there may be a sex-specific biological role for DHA in mobility [117]. However, other studies found no relationship with the relative change in muscular parameters [116], decline in gait speed [117,118], or decline in physical performance [118] after three and five years. As frequently encountered with longitudinal studies, the decline in the main outcome or the incidence of the condition being looked at may not have been sufficient to detect significant associations. Also, considering the limited evidence available, it can only be concluded that cross-sectional and longitudinal associations of plasma or erythrocyte *n*-3 PUFA concentrations and muscle mass or function remain uncertain. In future studies, analysis with EPA and DHA levels should consistently be reported, as detected relationships with total *n*-3 PUFA were driven by these specific fatty acids.

*Interventional studies.* One study previously reported a modest effect of a six-month, 1.2 g/day *n*-3 PUFA (720 mg EPA and 480 mg DHA) supplementation on gait speed in 126 seniors aged ≥65 years, but not on body composition and strength [119]. Three additional recent studies have tested daily 1.1–3.36 g EPA and DHA supplementation over three to six months. Following a six-month, 4 g/d *n*-3 PUFA supplementation (1.86 g EPA, 1.5 g DHA) compared to corn oil placebo, Smith et al. (2015) detected gains of 2.3 kg in handgrip strength (95% CI: 0.8, 3.7 kg) and of 3.6% (95% CI: 0.2, 7.0%) in thigh muscle volume in community-dwelling older adults (*n* = 60; aged 60 to 85 years) [120]. The authors estimated that these beneficial changes would result in a two to three-year prevention of muscle mass and function decline normally associated with aging. Similarly, Logan et al. (2015) reported a 4% increase (1.6 kg ± 0.7 kg, *p* = 0.01) in muscle mass as measured by bioelectrical impedance analysis BIA and a 7% improvement in the TUG in the intervention group (5 g/day fish oil; 2 g of EPA, 1 g of DHA) compared to an olive oil placebo after three months (*n* = 24 healthy women aged 60–76 years; those who consumed >1 meal of fish per week or took *n*-3 supplements were excluded) [121]. Yet, another three-month trial showed no effect of a 1.3 g/day *n*-3 PUFA supplementation (660 mg of EPA, 440 mg of DHA) on body composition (by BIA), TUG, handgrip strength, and gait speed in 53 older adults (age ≥65 years) at risk (1 SD below the mean ALM index of a reference population) or having low lean mass at baseline [122]. This absence of effect is possibly due to the low EPA and DHA doses provided and the relatively short duration of the intervention [119,122]. Although a biomarker measure of *n*-3 PUFA was not reported in the latter study, those that provided doses of 3.0 g and 3.36 g of EPA and DHA effectively increased serum and red blood cells’ *n*-3 PUFA concentrations after three [121] and six [120] months, respectively, versus no change in the control groups. The effect of doses between 1.1–3.0 g was not tested in untrained older adults.

It is of note that the methodologies of these trials used different approaches to body composition assessment, the duration of interventions, measures of *n*-3 PUFA status, and doses of supplements, which prevents direct comparison of studies. Future trials should report at least one measure of *n*-3 PUFA circulating levels as well as its changes to allow proper interpretation of the efficacy of the supplement [112]. Yet, a three-month intervention appears long enough to see improvements, but only doses ≥3.0 g/day showed a compelling increment in functional measures and muscle mass and volume [120,121]. While *n*-3 PUFA may prevent sarcopenia in healthy older adults, its effect in sarcopenic seniors and those losing autonomy remains to be investigated.

## 6. Combined Supplements of Protein, Leucine, Vitamin D, and *n*-3 PUFA

While increased protein intakes and supplements in leucine, vitamin D, and *n*-3 PUFA support potential gains in muscle mass and function when consumed individually, the combination of these nutrients may provide further benefits. Four recent, randomized, double-blind placebo-controlled studies tested a combined supplement of high-quality protein and vitamin D, without exercise intervention, on lean mass, strength, and physical performance [123,124,125,126]; two of them included added leucine to the mix [124,126], and one included *n*-3 PUFA, and creatine [125]. A 13-week multicenter study conducted in 380 sarcopenic older individuals aged ≥65 years with high disability risk found a beneficial effect of a 800 IU vitamin D, 3 g of leucine, and 20 g of whey protein supplement, given twice daily, on the chair-stand time (−1.01 s, 95% CI: −1.77, −0.19), but not on physical performance, mobility, and strength compared to the control group receiving an isocaloric placebo [126]. A very slight increase in ALM (0.17 kg, 95% CI: 0.004, 0.338) was reported, although it was within the 1.2% lean body mass measurement error by dual-energy X-ray absorptiometry DXA [127]. The same supplement was provided before breakfast only to 24 healthy older men, and similarly showed a modest gain in ALM (estimate difference of 0.37 kg) and leg lean mass after six weeks [124]. Interestingly, dietary protein intake was displaced by the supplement in the intervention group, as shown by the same total protein intake between groups when accounting for the supplement. The modest improvement in outcomes may be attributable to the change in meal protein intake distribution, as breakfast protein consumption was higher in the intervention group at the end of the trial, with an average of >25 g at each meal compared to ~10 g at breakfast, at baseline, and in the control group. Again, the supplement did not show any improvement on handgrip strength and physical performance. Bell et al. examined the effect of a similar multinutrient supplement that had 30 g of whey protein, 2.5 g of creatine, 500 IU of vitamin D, 400 mg of calcium, and 1.5 g of *n*-3 PUFA, 700 mg of EPA, 445 mg of DHA, and was consumed twice per day (1 h after breakfast and 1 h before going to bed) against a placebo of 22 g of maltodextrin, on strength and lean mass in 49 healthy older men (aged 73 ± 1 years) during six weeks alone and in combination with high-intensity interval trainings during the 12 following weeks [125]. After six weeks of supplementation alone, both lean mass and strength increased in the intervention group; the authors reported gains of 0.4 kg for ALM and 3% for the sum of isotonic strength, but the intervention was only superior to the control for the latter. Similarly, Bo et al. found an improvement in handgrip strength (1.91 ± 4.24 kg, *p* = 0.020) from a long, six-month intervention of 22 g of whey protein, 702 IU of vitamin D, and 109 mg of vitamin E consumed before breakfast and dinner, in 60 sarcopenic men and women aged 65–80 years (versus an isocaloric placebo). Although participants receiving the treatment did not gain muscle mass, a declining trend was seen in the control group, suggesting that the supplementation was protective of muscle mass loss over six months. The authors did not find any effect from the intervention on mobility and physical performance.

Importantly, none of these studies reported serious adverse effects from supplements. Combined supplements showed favorable effects on muscle mass, but may be more effective at stimulating MPS in healthy older adults, and promoting muscle mass maintenance in sarcopenic older adults. Their impact on muscle strength is inconsistent, and no improvement in physical performance was observed. Although isolating the effect of each nutrient from a combined supplement is impossible, providing high-quality proteins, leucine, vitamin D, and *n*-3 PUFA all together appears to be promising in the prevention of sarcopenia, while also being safe. Indeed, more longer-term (i.e., >six weeks) research of multinutrient supplementation involving populations at risk of greater muscle mass decline, such as frail individuals and those losing autonomy, is needed.

## 7. Conclusions

Proteins, leucine, vitamin D, and *n*-3 PUFAs may individually play a protective role in skeletal muscle health. Therefore, inadequate intakes of these nutrients could lead to several prejudicial conditions such as sarcopenia, frailty, loss of mobility and physical function, morbidity, and mortality. Groups of authors recommend protein intakes of 1.0–1.2 g/kg/day for older adults, which is higher than the currently established RDA, but may not always be achievable through dietary sources for some individuals with reduced appetite. The manifestation of anabolic resistance associated with aging explains, among other factors, the increase in protein requirements. In addition, an even distribution of proteins throughout the day may favor the reach of the anabolic threshold to maximally stimulate MPS. However, this effect remains to be confirmed by long-term intervention studies, and may differ between healthy and malnourished individuals. The effect of vitamin D on physical function seems to be essentially beneficial in individuals with prior vitamin D deficiency. Also, doses of 800–1000 IU/day appear to be more effective compared to lower doses. With respect to *n*-3 PUFAs, recent evidence suggests that EPA + DHA doses of ~3 g/day may have a positive impact on physical performance, muscle strength, and muscle mass in older adults, and this minimal amount may be required for beneficial effects when provided alone, i.e., not combined to other nutrients. Heterogeneity makes comparison of studies difficult, such that future studies should have standardized methods.

Key nutrient supplementation in older adults is of interest in the prevention of sarcopenia and frailty, since it is a simple, low-cost treatment approach without major side effects. Intervention studies testing a combined nutritional supplement are to be explored given the potentially additive effects of proteins, vitamin D, and *n*-3 PUFAs in the prevention of muscle mass and function loss.

## Abbreviations

MPS	Muscle protein synthesis
*n*-3 PUFA	*n*-3 polyunsaturated fatty acids
RDA	Recommended Dietary Allowance
ALM	Appendicular lean mass
MyoPS	Myobfibrillar protein synthesis
RCT	Randomized controlled trials
SPPB	Short Physical Performance Battery
VDR	Vitamin D receptor
EPA	Eicosapentaenoic acid
DHA	Docosahexaenoic acid
TUG	Timed-Up-and-Go
DXA	Dual-energy X-ray absorptiometry
BIA	Bioelectrical impedance
